# A Hydroxytricyanopyrrole-Based Fluorescent Probe for Sensitive and Selective Detection of Hypochlorous Acid

**DOI:** 10.3390/molecules27217237

**Published:** 2022-10-25

**Authors:** Chunhua Zeng, Zhengjun Chen, Mingyan Yang, Jiajia Lv, Hongyu Li, Jie Gao, Zeli Yuan

**Affiliations:** 1 Key Laboratory of Basic Pharmacology of the Ministry of Education and Joint International Research Laboratory of Ethnomedicine of the Ministry of Education, Zunyi Medical University, No. 6 West Xuefu Road, Xinpu District, Zunyi 563000, China; 2 Key Laboratory of Biocatalysis & Chiral Drug Synthesis of Guizhou Province, School of Pharmacy, Zunyi Medical University, No. 6 West Xuefu Road, Xinpu District, Zunyi 563000, China; 3 Guizhou International Scientific and Technological Cooperation Base for Medical Photo-Theranostics Technology and Innovative Drug Development, Zunyi Medical University, No. 6 West Xuefu Road, Xinpu District, Zunyi 563000, China

**Keywords:** reactive oxygen species, hypochlorous acid, fluorescence, hydroxytricyanopyrrole, intramolecular charge transfer

## Abstract

Hypochlorous acid (HOCl) is a reactive substance that reacts with most biomolecules and is essential in physiological and pathological processes. Abnormally elevated HOCl levels may cause inflammation and other disease responses. To further understand its key role in inflammation, HOCl must be detected in situ. Here, we designed a hydroxytricyanopyrrole-based small-molecule fluorescent probe (HTCP-NTC) to monitor and identify trace amounts of HOCl in biological systems. In the presence of HOCl, HTCP-NTC released hydroxyl groups that emit strong fluorescence covering a wide wavelength range from the visible to near-infrared region owing to the resumption of the intramolecular charge transfer process. Additionally, HTCP-NTC demonstrated a 202-fold fluorescence enhancement accompanied by a large Stokes shift and a low detection limit (21.7 nM). Furthermore, HTCP-NTC provided a rapid response to HOCl within 18 s, allowing real-time monitoring of intracellular HOCl. HTCP-NTC exhibited rapid kinetics and biocompatibility, allowing effective monitoring of the exogenous and endogenous HOCl fluctuations in living cells. Finally, based on fluorescence imaging, HTCP-NTC is a potential method for understanding the relationship between inflammation and HOCl.

## 1. Introduction

As a class of endogenous metabolites, reactive oxygen species (ROS), are involved in various physiological and pathological processes [1]. Abnormally elevated ROS levels can impair basic cellular functions and are closely associated with the onset and progression of various diseases, including inflammation, cancer, cardiovascular disease, and neurodegenerative diseases [1,2,3,4,5,6,7]. Hypochlorous acid (HOCl), which is an ROS exhibiting high reactivity, is generated through a peroxidation reaction between hydrogen peroxide and chloride ions catalyzed by myeloperoxidase [8]. Normally, HOCl is a vital oxidizing agent in the immune system that aids in the defense against pathogenic microorganisms such as bacteria and viruses [9,10,11]. However, HOCl overexpression can oxidize amino acids, peptides, proteins, phospholipids, and deoxyribonucleic acid, causing cellular dysfunction and tissue damage [12,13,14]. Consequently, many diseases, such as atherosclerosis and rheumatoid arthritis, are closely related to HOCl overproduction [15,16,17]. As a result, it is essential to develop highly selective and sensitive fluorescent probes for the rapid detection of HOCl fluctuations in biological systems.

Among various kinds of analytical methods, fluorescent probes can detect HOCl in biological organisms in real-time and in situ, so several fluorophore-based fluorescent probes, including coumarin [18,19,20,21,22,23], BODIPY [24,25,26,27,28,29], rhodamine [30,31,32,33,34,35], naphthalimide [36,37,38,39,40,41], etc., have been developed for imaging HOCl in vitro and in vivo [42,43,44,45,46,47,48,49,50,51,52]. The detection mechanism is primarily the oxidation reaction of HOCl with *p*-alkoxyaniline, *p*-methoxyphenol, oxime, hydrazine, electron-deficient C=C bonds, and S/Se/Te, resulting in fluorescence signals “on” or “off” [53,54]. Although these probes exhibit excellent properties, they also have disadvantages such as low sensitivity, slow response rate, and poor biocompatibility. Consequently, the development of small molecule fluorescent probes with good biocompatibility is necessary for the fast detection of HOCl in living cells.

**Scheme 1:**
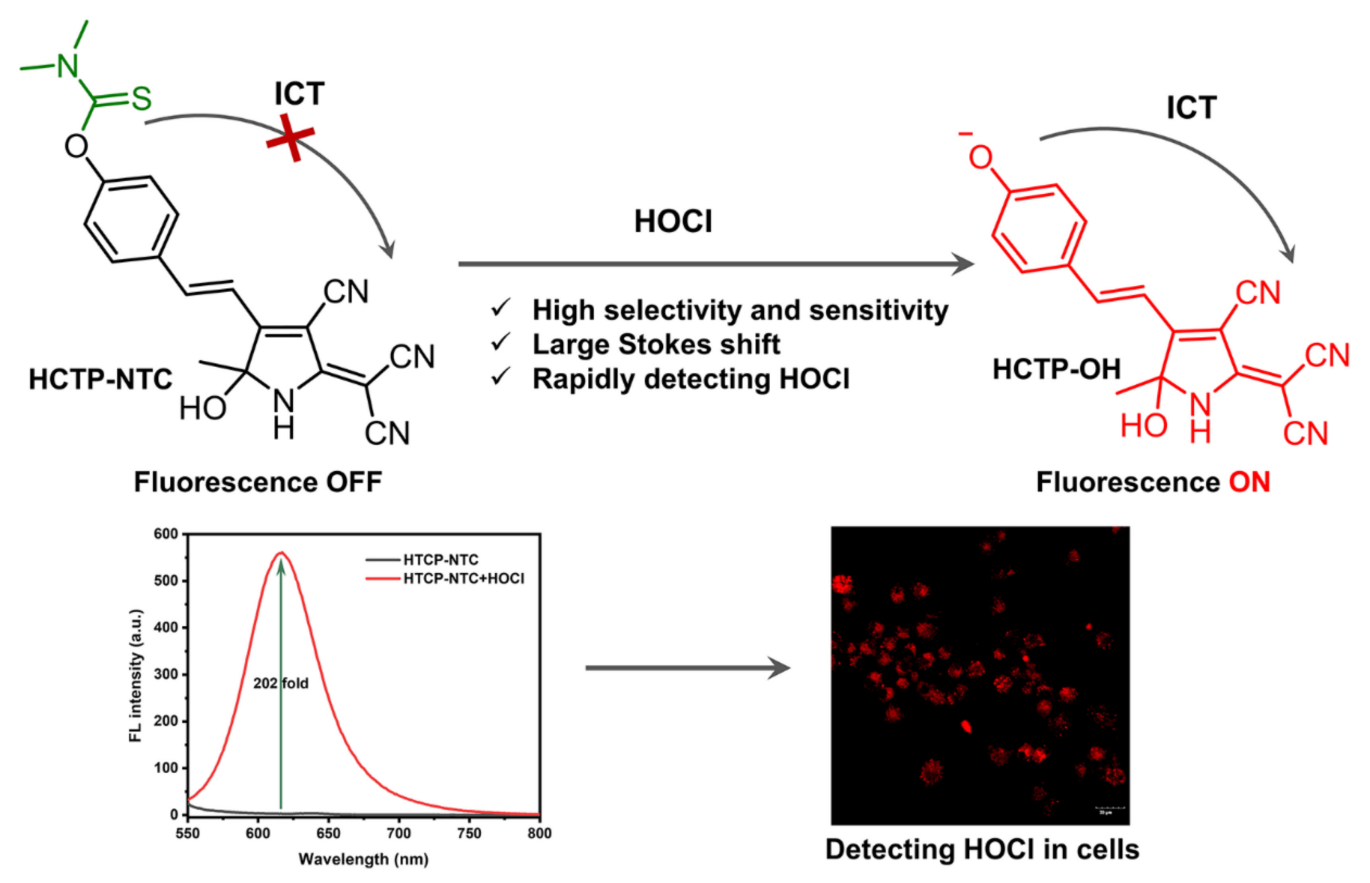
Schematic illustration of the HTCP-NTC probe response to HOCl.

In this study, a small molecule fluorescent probe, HTCP-NTC, based on intramolecular charge transfer (ICT) was designed for HOCl detection (Scheme 1). Hydroxytricyanopyrrole (HTCP), a cyano-rich heterocyclic compound, acted as an electron acceptor. Additionally, phenol was selected as an electron donor with a phenolic hydroxyl position modification dimethylthiocarbamate serving as a specific response site for HOCl. In the absence of HOCl, the HTCP-NTC fluorescence signal was weak, and the ICT effect was suppressed. However, in the presence of HOCl, the response group of HTCP-NTC was removed and the ICT effect was activated, at which point the fluorescence signal was rapidly enhanced, resulting in specific and rapid detection of HOCl. Notably, HTCP-NTC was successfully applied to HOCl imaging in living cells. To the best of our knowledge, HTCP-NTC is the first probe to achieve a fluorescence response based on the HTCP receptor.

## 2. Results and Discussion

### 2.1. Design and Synthesis of HTCP-NTC

**Scheme 2:**
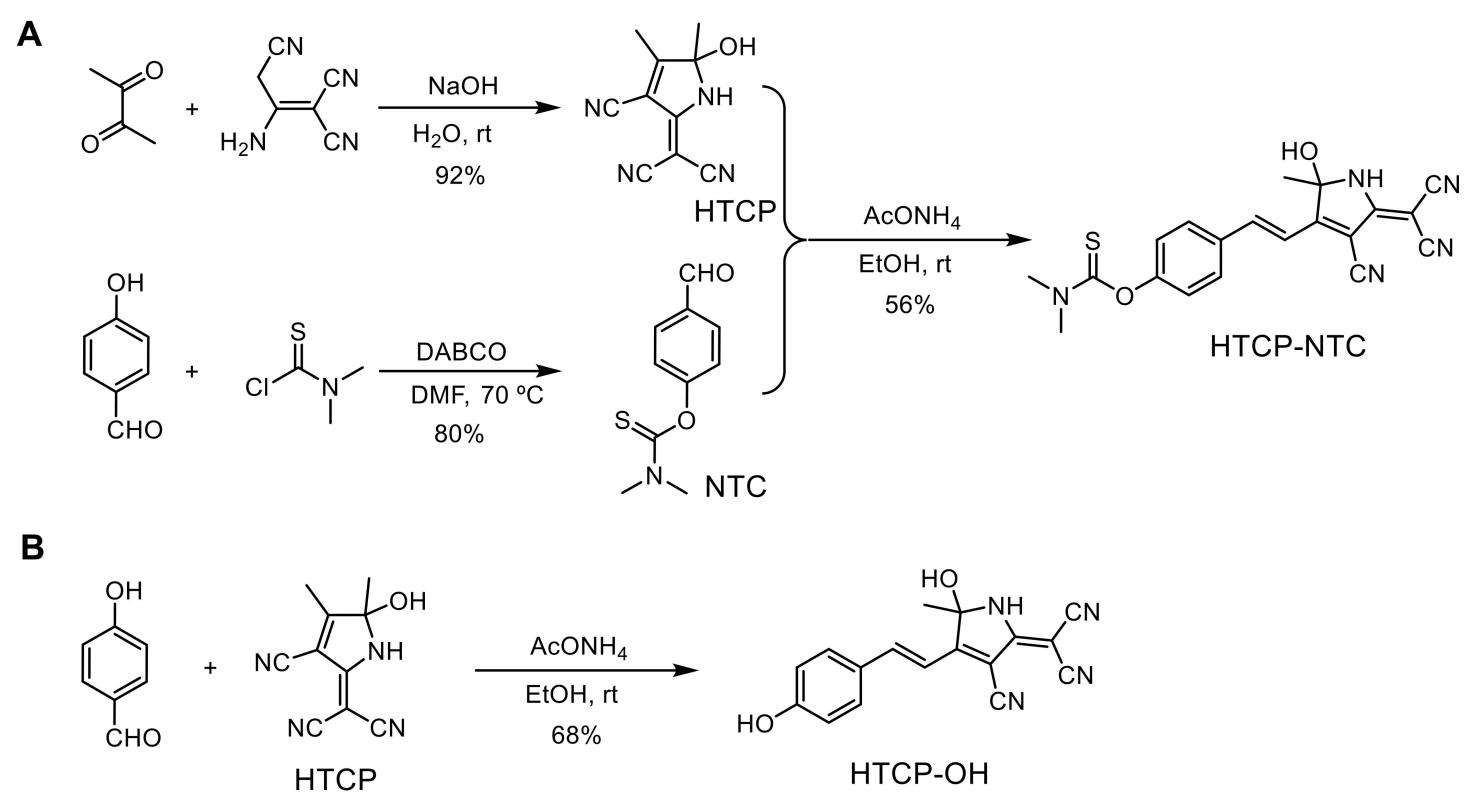
The synthetic route of the fluorescent probe HTCP-NTC (**A**) and HTCP-OH (**B**). DABCO: triethylenediamine.

To achieve a specific response to HOCl, we designed HTCP-NTC, an ICT-based fluorescent probe. The HTCP moiety was considered as an electron acceptor and was an analog of the tricyanofuran chromophores. Phenol was selected as an electron donor, the phenolic hydroxyl group of which could be used to modify specific groups to mask the fluorescence emission by suppressing the ICT processes. The HTCP and phenol groups combine to form an electron acceptor-π-electron donor type of NIR fluorescent molecule. *N*,*N*-dimethylthiocarbamate served as a response site for the sensitive and specific detection of HOCl. HTCP was synthesized from 2,3-butanedione and 2-aminoprop-1-ene-1,1,3-tricarbonitrile (92% yield) [55,56,57]. Furthermore, *N*,*N*-dimethylthiocarbamate was modified to the phenolic hydroxyl position of 4-hydroxybenzaldehyde to obtain NTC (80% yield). Finally, the HTCP-NTC was prepared via the Knoevenagel condensation reaction of HTCP and NTC (56% yield) (Scheme 2A). Additionally, the HTCP-OH was synthesized to verify the mechanism of HTCP-NTC response to HOCl. HTCP-OH was synthesized via the Knoevenagel condensation reaction of HTCP and 4-hydroxybenzaldehyde (80% yield) (Scheme 2B). The chemical structures of HTCP-NTC and HTCP-OH were characterized by nuclear magnetic resonance (NMR) spectroscopy and high-resolution mass spectrometry (HRMS) (Figures S1–S10 from Supplementary Materials).

### 2.2. The Spectroscopic Properties of HTCP-NTC

**Figure 1:**
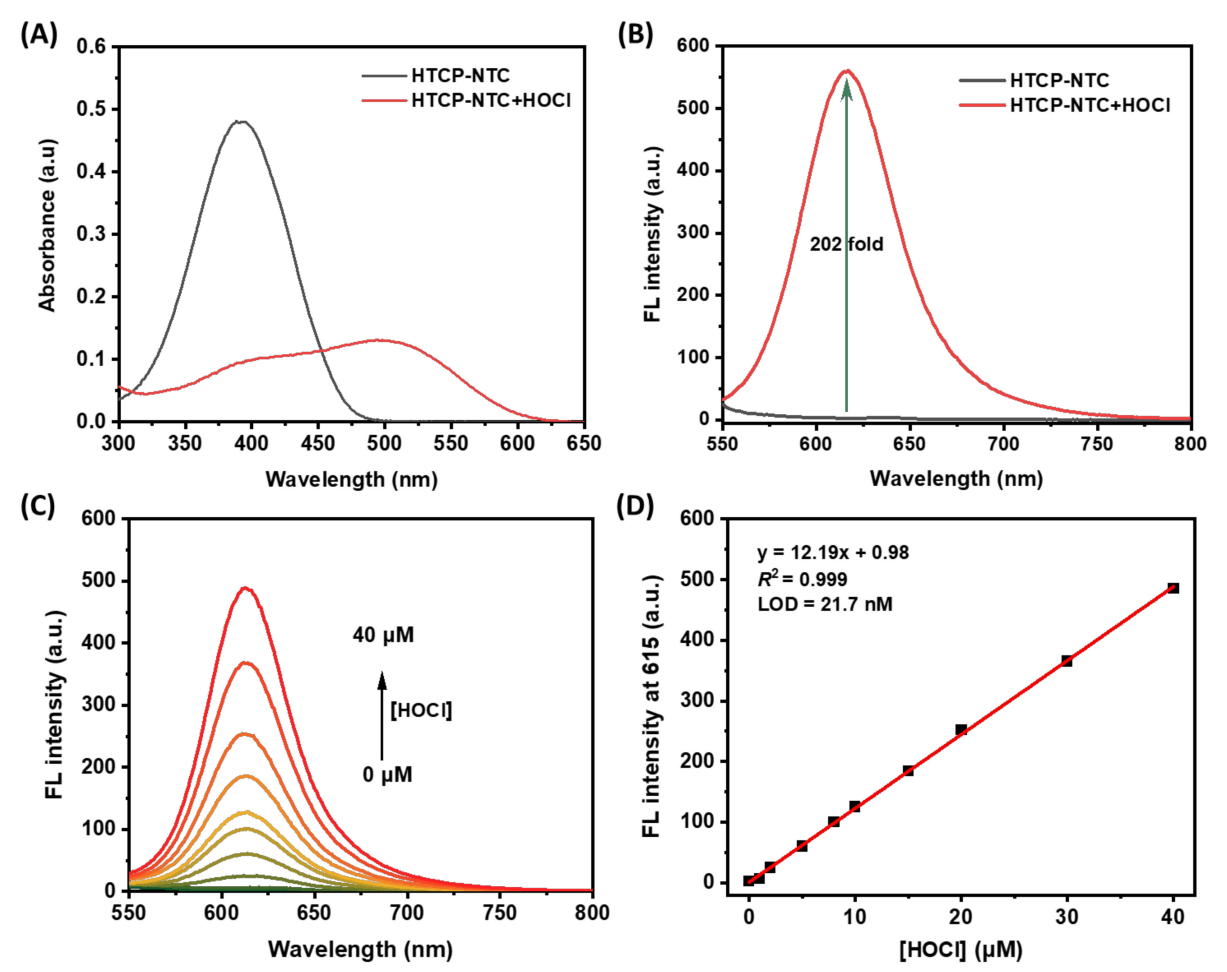
The changes of absorption (**A**) and fluorescence (**B**) spectra of HTCP-NTC (10 μM) in the presence and absence of HOCl (100 μM). (**C**) The changes in fluorescence spectra of HTCP-NTC (10 μM) with increasing concentration of HOCl (0–40 μM). (**D**) The fitting curve of the fluorescence intensity at 615 nm of HTCP-NTC (10 μM) vs. HOCl concentration (0–40 μM). Condition: PBS buffer (10 mM, pH = 7.4, containing 1% EtOH), λ_ex_ = 520 nm.

All spectral properties of HTCP-NTC were evaluated in PBS buffer (10 mM, pH 7.4, containing 1% EtOH). HTCP-NTC exhibited the maximum absorption at 391 nm. After adding HOCl to the HCTP-NTC solution, a new absorption peak at 502 nm appeared in the absorption spectrum with a red shift of 111 nm (Figure 1A). Furthermore, HTCP-NTC performance was focused on measuring the response to HOCl. In the absence of HOCl, HTCP-NTC showed almost no fluorescence emission. However, the fluorescence signal of HTCP-NTC was activated after the addition of HOCl (100 μM), exhibiting a maximum emission intensity at 615 nm with a 202-fold signal enhancement relative to the absence of HOCl. Furthermore, the fluorescence quantum yields of HTCP-NTC and HTCP-OH were measured to be 0.35% and 0.14%, using ethidium bromide in water as reference (2.3%) [58]. Notably, HTCP-NTC also exhibits a large Stokes shift (113 nm) after the hypochlorite response, which is beneficial for subsequent fluorescence imaging of the cells (Figure 1B). This phenomenon should be attributed to ability of HOCl to rapidly react with *N*,*N*-dimethylthiocarbamate and thus activate the fluorescence signal, indicating that HTCP-NTC could be used for HOCl fluorescence detection. Inspired by the above results, we measured the fluorescence response of HTCP-NTC to various concentrations of HOCl (Figure 1C). There is a good linear relationship between the fluorescence intensity at 615 nm and the concentration of HOCl (FL intensity = 12.19 [HOCl] + 0.98, *R*^2^ = 0.999) (Figure 1D). The limit of detection (LOD) was measured to be 21.7 nM based on 3σ/*k* (σ: standard deviation; *k*: slope), indicating that HTCP-NTC can identify trace amounts of HOCl. The relative LOD of our probe compared to other probes reported suggests that HTCP-NTC has a high potential for detecting HOCl (Table S1 from Supplementary Materials).

### 2.3. Selectivity of the HTCP-NTC

**Figure 2:**
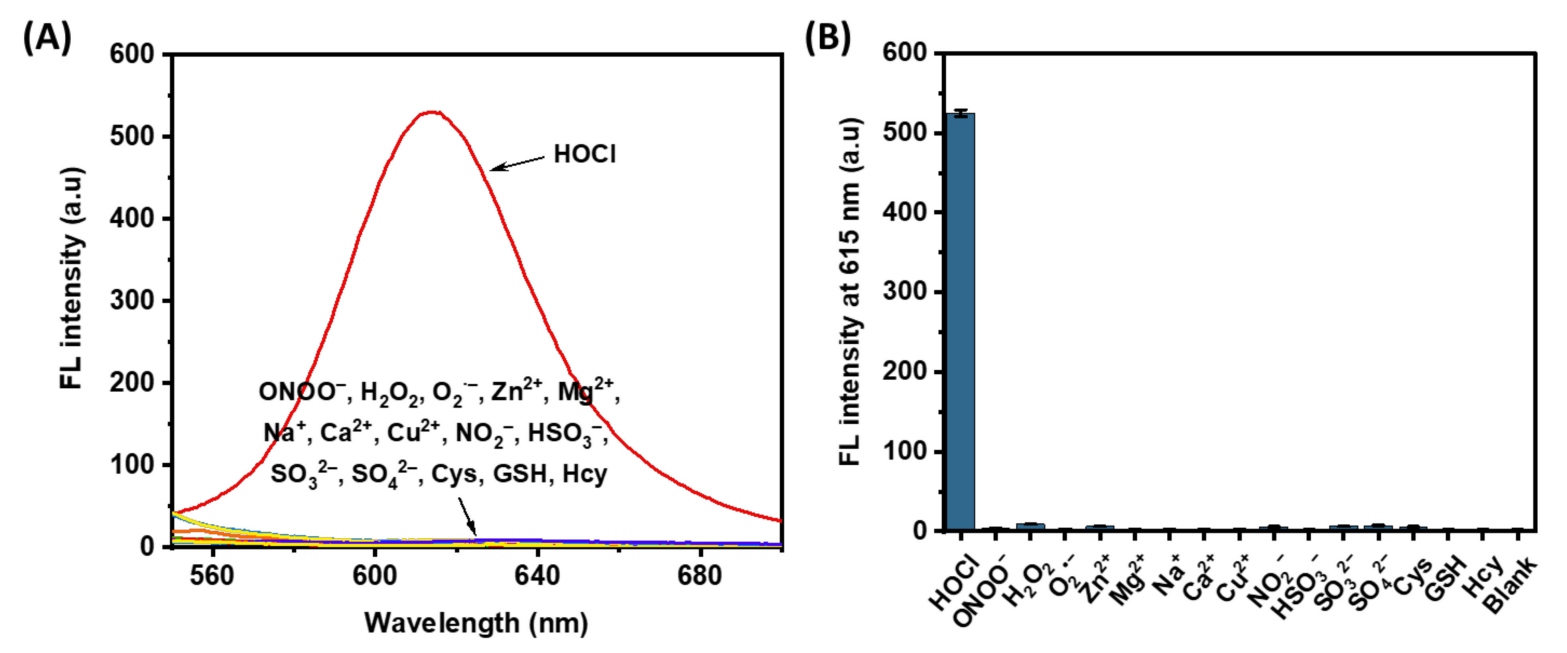
(**A**) Fluorescence emission spectra of HTCP-NTC (10 μM) after 15 min incubation with HOCl (50 μM), reactive species (100 μM), ions (100 μM), and biothiols (100 μM) in PBS buffer (10 mM, pH = 7.4, containing 1% EtOH). (**B**) The fluorescence intensity of HTCP-NTC (10 μM) for various species. “Blank” represented HTCP-NTC solution without the addition of any substance.

High selectivity is critical for ideal fluorescent molecules. The response of the probe HTCP-NTC to various common physiological substances was examined, including various reactive substances (ONOO^−^, H_2_O_2_, and O_2_^·−^), common ions (Zn^2+^, Mg^2+^, Na^+^, Ca^2+^, Cu^2+^, NO_2_^−^, HSO_3_^−^, SO_3_^2−^, and SO_4_^2−^), and biothiols (cysteine (Cys), glutathione (GSH), and homocysteine (Hcy)). According to the previous report [59], only HOCl led to a significant enhancement of the fluorescence signal as the HTCP-NTC was converted to HTCP-OH fluorophores, further indicating that the probe has excellent selectivity for HOCl (Figure 2).

### 2.4. Reaction Kinetics and Environment Effects

**Figure 3:**
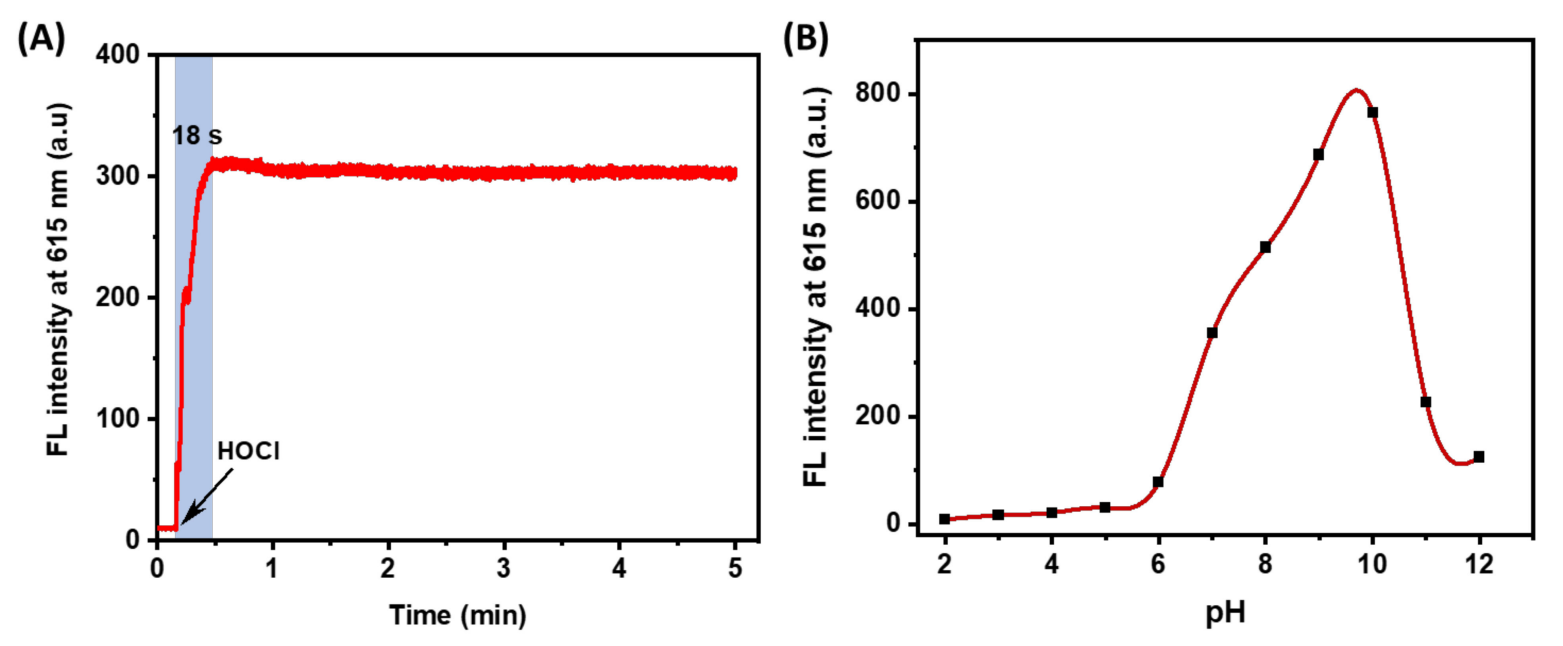
(**A**) The kinetic curve of HTCP-NTC (10 μM) reaction with HOCl (20 μM) in PBS buffer (10 mM, pH = 7.4, containing 1% EtOH). (**B**) Fluorescence intensity of HTCP-NTC (10 μM) HOCl (20 μM) at 615 nm in PBS buffer solution at different pH (10 mM, containing 1% EtOH).

The speed response of the probe to the substance determines the detection efficiency. Therefore, we investigated the reaction kinetics of HTCP-NTC in the presence of HOCl. The fluorescence intensity at 615 nm was measured for about 5 min following the addition of HOCl (Figure 3A). Notably, the fluorescence signal reached a maximum value (approximately 307) within 18 s and remained constant until the end of the measurement, indicating that HTCP-NTC could respond quickly to HOCl.

Furthermore, according to previous reports [60,61], the structural characteristics of HTCP-NTC and HTCP-OH may be environmentally sensitive, and thus their absorption and emission spectra were investigated under different pH, viscosity, and polarity conditions. First, the effect of pH on HTCP-NTC was evaluated. The fluorescence intensity of HTCP-NTC with HOCl at 615 nm was measured from pH 2 to pH 12 (Figure 3B). The results showed that HTCP-NTC produced a low fluorescence signal after the addition of HOCl at pH 2–6. The fluorescence signal was significantly enhanced from pH 7–10, and the fluorescence intensity increased with increasing pH. HTCP-OH has a pKa value of 7.9 (Figures S11 and S12 from Supplementary Materials), thus HTCP-NTC exhibits a weak fluorescence signal at low pH (2–6) and a strong fluorescence signal at high pH (7–10). The fluorescence intensity of HTCP-NTC decreases sharply at pH 11–12, for the HTCP moiety undergoing ring-opening [62]. Indeed, HTCP-NTC was affected by pH when detecting HOCl. Afterward, the changes of absorption and fluorescence spectra for HTCP-NTC and HTCP-OH were studied in different viscosity (PBS 7.4/glycerol system) and polar environments (various solvents), respectively. The HTCP-NTC and HTCP-OH all exhibited enhanced fluorescence signals with increasing viscosity (Figure S13 from Supplementary Materials), mainly due to the high viscosity environment limited molecular rotation. In different polar environments, HTCP-NTC exhibited a lower fluorescence signal, except in toluene (Figure S14 from Supplementary Materials). In contrast, HTCP-OH exhibited a stronger polarity sensitivity (Figure S14D from Supplementary Materials), which is mainly attributed to the presence of strong ICT. According to the above results, HTCP-NTC is still a promising fluorescent probe under fixed pH conditions (especially for pH 7–10).

### 2.5. Mechanism of HTCP-NTC Detection of HOCl

**Scheme 3:**
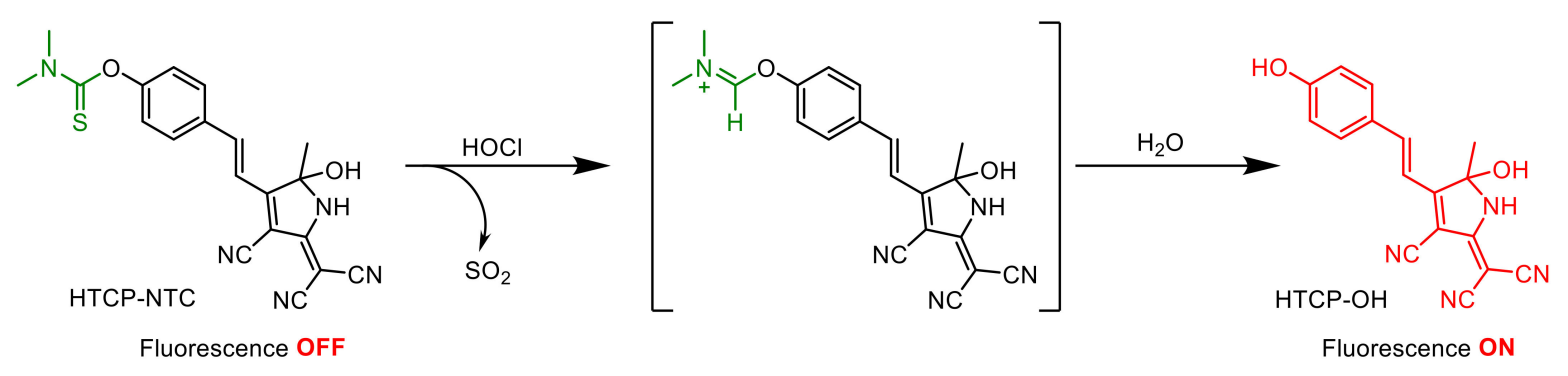
Proposed HOCl signaling mechanism of HTCP-NTC.

The fluorescence response of HTCP-NTC to HOCl was based on an ICT mechanism (Scheme 3). Before the reaction with HOCl, the recognition group of HTCP-NTC had a weak electron donating ability, inhibiting the ICT effect and the fluorescence emission was weak. The recognition group of HTCP-NTC was removed by a hydrolysis reaction, after interaction with HOCl [63,64,65]. Subsequently, the generated hydroxyl and cyano groups could act as electron donors (D) and electron acceptors (A), restoring the intramolecular push–pull electron (D-π-A) system. Because of the presence of strong ICT effects, an intense red fluorescence emission was generated for the determination of HOCl. Additionally, the response mechanism of HTCP-NTC to HOCl was further evaluated by using high-performance liquid chromatography and spectra. The retention times of HTCP-NTC and HTCP-OH were 14.47 and 12.75 min, respectively. As expected, a small peak appeared after the addition of HOCl with the same retention time (12.79 min) as HTCP-OH (Figure S15 from Supplementary Materials). The spectral characteristics of reaction mixtures for HTCP-NTC with HOCl were consistent with those of HTCP-OH in the absorption, emission and excitation spectra (Figure S16 from Supplementary Materials). The results determined that HTCP-NTC-generated HTCP-OH with HOCl.

### 2.6. Fluorescence Imaging of HOCl in Living Cells

**Figure 4:**
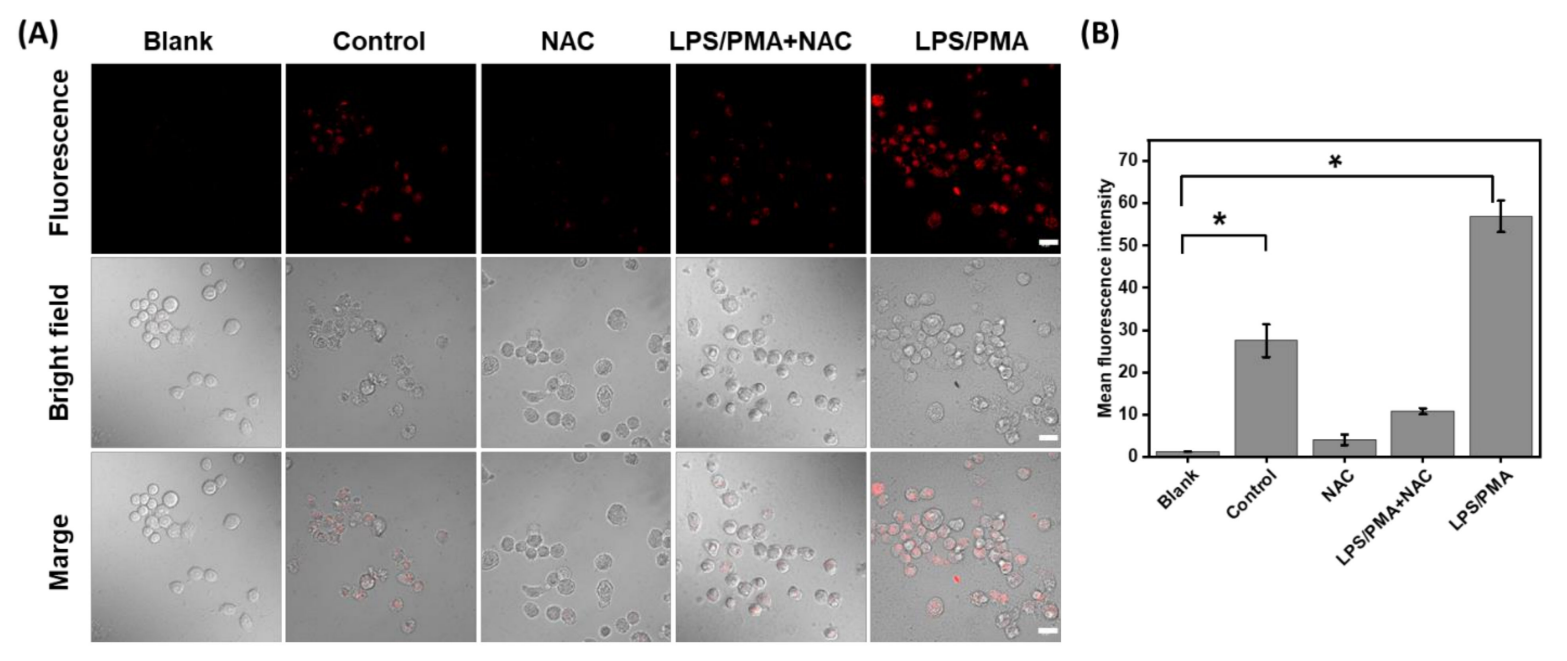
(**A**) Fluorescence imaging of endogenous HOCl in RAW264.7 cells by a confocal laser scanning microscope (CLSM). “Blank”: the cells without any treatment; “Control”: the cells were treated by HTCP-NTC (2.5 μM); “NCA”: the cells were pretreated with NAC (1 mM) for 1 h and then incubated with HTCP-NTC (2.5 μM); “LPS/PMA+NAC”: the cells were pretreated with LPS (5 µg/mL) for 12 h, PMA (5 µg/mL) for 4 h, and NAC (1 mM) for 1 h, and then incubated with HTCP-NTC (2.5 μM); “LPS/PMA”: the cells were pretreated with LPS (5 µg/mL) for 12 h and PMA (5 µg/mL) for 4 h, and then incubated with HTCP-NTC (2.5 μM). λ_ex_ = 488 nm, λ_em_ = 600–700 nm. Scale bar = 20 μm. (**B**) Fluorescence intensity statistics of each group of cells. * *p* < 0.05.

Inspired by the positive performance of HTCP-NTC, we further used HTCP-NTC to image and monitor endogenous HOCl in living cells. The cytotoxicity of HTCP-NTC was first evaluated in RAW 264.7 cells (murine macrophage cells) by using the 3-(4,5-dimethylthiazol-2-yl)-2,5-diphenyltetrazolium bromide (MTT) assay. The cell viability rate was greater than 90% after incubation of various concentrations of HTCP-NTC (0–20 μM) with RAW 264.7 cells for 24 h (Figure S17 from Supplementary Materials). Imaging of endogenous HOCl was performed in RAW 264.7 cells for the cells that could produce high levels of HOCl after stimulation with lipopolysaccharide (LPS) and foponol-12-myristate-13-acetate (PMA) [66,67,68]. Untreated RAW 264.7 cells incubated with HTCP-NTC revealed a weak fluorescence signal (Figure 4A). However, the fluorescence signal of RAW 264.7 cells pretreated with LPS/PMA was about twofold higher than that of untreated cells, indicating an increase in HOCl (Figure 4A). *N*-acetylcysteine (NAC) was selected as the scavenger. A significant decrease in fluorescence intensity was observed when NAC was added to untreated and treated cells, indicating a decrease in intracellular hypochlorite levels (Figure 4). The above results demonstrate the ability of HTCP-NTC to monitor HOCl levels in endogenous living cells.

## 3. Materials and Methods

### 3.1. Reagents and Apparatus

All reaction reagents were purchased from Energy Chemical (Shanghai, China) and used directly unless otherwise specified. All the solvents used in the reactions are commercially available super-dry solvents (Energy Chemical). The silica gel with a mesh of 200–300 was obtained from Shanghai Titan Scientific General-Reagent (Shanghai, China). The eluents were also produced by General-Reagent. The ^1^H NMR and ^13^C NMR spectra were recorded at room temperature by using an Agilent 400 DD2 spectrometer (Santa Clara, CA, USA). The HRMS was measured by using Thermo Scientific Q Exactive Orbitrap Mass Spectrometers (Franklin, MA, USA). The absorption and fluorescence spectra were obtained by using a Shimadzu UV-2600i UV-Vis spectrophotometer (Kyoto, Japan) and an Agilent Cary Eclipse fluorescence spectrometer (Santa Clara, CA, USA), respectively. The absorbance in MTT assays was measured by using a Thermo Fisher Multiskan FC microplate reader (Franklin, MA, USA). Cellular fluorescence imaging was performed by using an Olympus Fluoview FV3000 CLSM (Tokyo, Japan).

### 3.2. Synthesis of the HTCP

HTCP was prepared following previous literature [55,56,57] with slight modifications. We added 2,3-butanedione (2.64 g, 20 mmol) and 2-aminoprop-1-ene-1,1,3-tricarbonitrile (1.72 g, 20 mmol) to a round bottom flask followed by water (40 mL). The above solution was thoroughly stirred, and then NaOH solution (1 mL, 0.5 mol/L) was added. The mixture was stirred for 2 h at room temperature. After the completion of the reaction, the mixture was cooled to 0–5 °C, and the precipitated product was filtered. The precipitate was washed with dichloromethane and dried to give a white solid, with a 92% yield (^1^H NMR (400 MHz, CD_3_OD) *δ* 2.27 (s, 3H), 1.54 (s, 3H); ^13^C NMR (101 MHz, CD_3_OD) *δ* 178.4, 160.8, 113.8, 113.1, 110.4, 104.5, 93.9, 45.9, 21.8, 11.6).

### 3.3. Synthesis of the NTC

NTC was prepared following previous literature [63]. 4-hydroxybenzaldehyde (244 mg, 2 mmol), dimethylthiocarbamoyl chloride (307 mg, 2.5 mmol), and DABCO (392 mg, 3.5 mmol) were dissolved in DMF (10 mL). The mixture was heated to 70 °C while being stirred. The reaction process was detected by using TLC. When the reaction was completed, the reaction mixture was extracted sequentially with ethyl acetate and saturated sodium chloride. The product was purified by using column chromatography with petroleum ether/dichloromethane (1:3, *v*/*v*) as the eluent to yield NTC as a white solid (334 mg, 80%) (^1^H NMR (400 MHz, CDCl_3_) *δ* 10.00 (s, 1H), 7.92 (m, 2H), 7.24 (d, J = 8.4 Hz, 2H), 3.45 (s, 3H), 3.36 (s, 3H);^13^C NMR (101 MHz, CDCl_3_) *δ* 191.0, 186.6, 158.5, 134.0, 130.9, 123.7, 43.3, 38.9).

### 3.4. Synthesis of HTCP-NTC

HTCP (0.2 g, 1 mmol), NTC (313 mg, 1.5 mmol), and dried ammonium acetate (77 mg, 1 mmol) were mixed with anhydrous ethanol (5 mL). The mixture was stirred at room temperature for 6 h. Following the reaction completion, the solvent was removed under vacuum. The residue was purified by column chromatography with dichloromethane/methanol (100:1, *v*/*v*) as the eluent, yielding HTCP-NTC as a yellow solid (219 mg, 56%) (^1^H NMR (400 MHz, DMSO-*d6*) *δ* 10.61 (s, 1H), 7.87 (m, 2H), 7.83 (s, 1H), 7.19 (dd, *J* = 9.1, 2.3 Hz, 2H), 7.13 (s, 1H), 3.37 (s, 6H), 1.71 (s, 3H); ^13^C NMR (101 MHz, DMSO-*d6*) *δ* 186.2, 170.8, 160.6, 156.4, 145.0, 132.6, 130.3, 124.3, 116.6, 115.6, 114.4, 112.1, 100.9, 94.1, 62.7, 55.4, 43.3, 25.9. HR-MS: *m*/*z* C_20_H_18_N_5_O_2_S^+^ calcd, 392.1176; found [M+H]^+^, 392.1186; C_20_H_21_N_6_O_2_S^+^ calcd, 409.1441; found [M+NH_4_]^+^, 409.1442).

### 3.5. Synthesis of HTCP-OH

HTCP (1 g, 5 mol), 4-hydroxybenzaldehyde (1.22 g, 10 mol), and dried ammonium acetate (385 mg, 5 mmol) were mixed with anhydrous ethanol (15 mL). The mixture was stirred for 6 h at room temperature. Following the reaction completion, the reaction solution was poured into petroleum ether (300 mL). The mixture was filtered and washed with petroleum ether to obtain HTCP-OH as a red solid (1.03 g, 68%) (^1^H NMR (400 MHz, DMSO-*d6*) *δ* 10.47 (s, 1H), 10.42 (s, 1H), 7.79 (d, *J* = 16.2 Hz, 1H), 7.69 (d, *J* = 8.8 Hz, 2H), 7.15 (d, *J* = 0.9 Hz, 1H), 6.95 (d, *J* = 16.1 Hz, 1H), 6.87 (d, *J* = 8.7 Hz, 2H), 1.68 (s, 3H); ^13^C NMR (101 MHz, DMSO-*d6*) *δ* 171.6, 161.8, 160.9, 146.8, 131.8, 126.3, 116.8, 115.8, 114.6, 113.2, 112.4, 98.2, 93.9, 44.2, 26.2; HR-MS: *m*/*z* C_17_H_13_N_4_O_2_^+^ calcd, 305.1034; found [M+H]^+^, 305.1039; C_17_H_16_N_5_O_2_^+^ calcd, 322.1299; found [M+NH_4_]^+^, 322.1307).

### 3.6. Spectrum of HTCP-NTC with HOCl

Given that HTCP-NTC was used to detect HOCl in live cells, the spectroscopic property test was conducted in PBS (10 mM, pH = 7.4). As a co-solvent, 1% ethanol was used. HTCP-NTC stock solution was prepared with ethanol. HTCP-NTC stock solution and PBS buffer were added to an Eppendorf tube, and the ethanol ratio was maintained at 1%. After the solution was mixed homogeneously, HOCl was added, and then the fluorescence or absorption spectrum was recorded.

### 3.7. Determination of the Fluorescence Quantum Yield

Fluorescence quantum yields were determined by using ethidium bromide in water as reference (2.3%) [58]. The fluorescence quantum yield was calculated by using the following equation: *Φ*_f(X)_ = *Φ*_f(S)_ × (*I* × (1 − 10^−*A*s^))/(*I*_S_ (1 − 10^−*A*x^)), where *Φ*_f_ is the fluorescence quantum yield, *A* is the absorbance at the excitation wavelength, *I* is the area under the emission curve, and subscript S and X refer to the reference and to the sample.

### 3.8. Determination of LOD

The LOD value was calculated by using a fluorescence titration method based on previous research [69]. The fluorescence intensity of HTCP-NTC at 615 nm was measured 11 times, and then σ was calculated. The linear relationship was fitted by the fluorescence intensity at 615 nm and the HOCl concentration. *k* can be determined from the standard curve. Finally, the LOD value was calculated by using the formula LOD = 3σ/*k*.

### 3.9. Reaction Kinetics of HTCP-NTC with HOCl

The HTCP-NTC solution (10 μM, PBS buffer 10 mM, pH = 7.4, containing 1% EtOH) was prepared in a fluorescent cuvette. The fluorescence spectrophotometer was set to the kinetic mode (λ_ex_ = 520 nm, λ_em_ = 615 nm). The HOCl solution was quickly added to the above mixture while vigorously stirring, and then the reaction kinetics was recorded for 5 min.

### 3.10. Cell Culture and Cytotoxicity Assays

RAW 264.7 cells were cultured in Dulbecco’s Modified Eagle Medium supplemented with 10% fetal bovine serum (FBS), 1% antibiotics (penicillin and streptomycin sulfate, 100 U/mL), and incubated at 37°C with 5% CO_2_ in a cell incubator.

The toxicity of HTCP-NTC to RAW 264.7 cells was determined bu using the MTT method. The cells were inoculated in 96-well plates (5000 cells per well) for 24 h. Subsequently, the cells were washed three times with PBS and incubated with various HTCP-NTC concentrations. The cells without any treatment served as controls. After incubation for 24 h, the cell culture medium was replaced with fresh medium. Then, MTT solution was added to each well and incubated for 4 h. Finally, the medium was drained and DMSO (100 µL) was added. The absorbance was measured at 490 nm by using a microplate reader (Multiskan FC, Thermo Fisher, USA). The cell viability was calculated by using the following formula: cell viability = ((OD490_HTCP-NTC_ − OD490_Blank_)/(OD490_control_ − OD_Blank_)) × 100%. All the tests were conducted three times.

### 3.11. Confocal Fluorescence Imaging

Fluorescence imaging was performed on an Olympus Fluoview FV3000. The cells were placed in confocal Petri dishes and then incubated for 8 h before fluorescence imaging. The confocal parameters were set to λ_ex_ = 488 nm and λ_em_ = 600–700 nm.

## 4. Conclusions

In summary, we developed a new fluorescent probe, HTCP-NTC, that can be used to track the intracellular HOCl levels in living cells. This probe has excellent specificity, good sensitivity, and rapid response to HOCl. Notably, the probe has a low detection limit and good linearity for HOCl, providing a reliable basis for quantitative HOCl analysis. HTCP-NTC demonstrated good biocompatibility in vitro. Additionally, the proposed HTCP-NTC has been used to image and detect HOCl levels in living cells. In the future, HTCP-NTC or its derivatives are expected to be used for studying the relationship between disease models such as inflammation and HOCl.

## Data Availability

The data presented in this study are available in the Supplementary Materials.

## Supplementary Materials

The following supporting information can be downloaded at: https://www.mdpi.com/article/10.3390/molecules27217237/s1, Figures S1, S3, S5 and S8: ^1^H NMR spectra of HTCP, NTC, HTCP-NTC and HTCP-OH; Figures S2, S4, S6 and S9: ^13^C NMR spectra of HTCP, NTC, HTCP-NTC and HTCP-OH; Figures S7 and S10: HRMS of HTCP-NTC and HTCP-OH; Figure S11: Fluorescence emission spectra of HTCP-NTC with HOCl at different pH; Figure S12: The pKa measurement of HTCP-OH; Figure S13: The absorption and fluorescence changes for HTCP-NTC and HTCP-OH in various viscosity; Figure S14: The absorption and fluorescence changes for HTCP-NTC and HTCP-OH in solvents of different polarity; Figure S15: HPLC analysis of HTCP-NTC response with HOCl; Figure S16: The absorption, fluorescence emission and excitation spectra of HTCP-OH and the reaction mixtures for HTCP-NTC with HOCl; Figure S17: The cell viability of RAW264.7 after 24 h incubation with HTCP-NTC; Table S1: The summary of photophysical properties of reported similar probes. References [59,70,71,72,73,74,75,76,77] are cited in the Supplementary Materials.

## Sample Availability

Samples of all compounds are available from the authors.
