# Supplementary material for: A Hydroxytricyanopyrrole-Based Fluorescent Probe for Sensitive and Selective Detection of Hypochlorous Acid
Source: Molecules. 2022 Oct 25;27(21):7237. doi: 10.3390/molecules27217237 (PMC9656901; doi:10.3390/molecules27217237)
Supplement: Supplementary file 1 [file molecules-27-07237-s001.zip › molecules-1981880-supplementary.pdf]

Molecules

Supplementary Materials

A Hydroxytricyanopyrrole-Based Fluorescent Probe for Sensitive and Selective Detection of Hypochlorous Acid

Chunhua Zeng [1,2,3,#], Zhengjun Chen [1,2,3,#], Mingyan Yang [1,2,3], Jiajia Lv [1,2,3,], Hongyu Li [1,2,3,], Jie Gao [1,2,3,*] and Zeli Yuan [1,2,3,*]

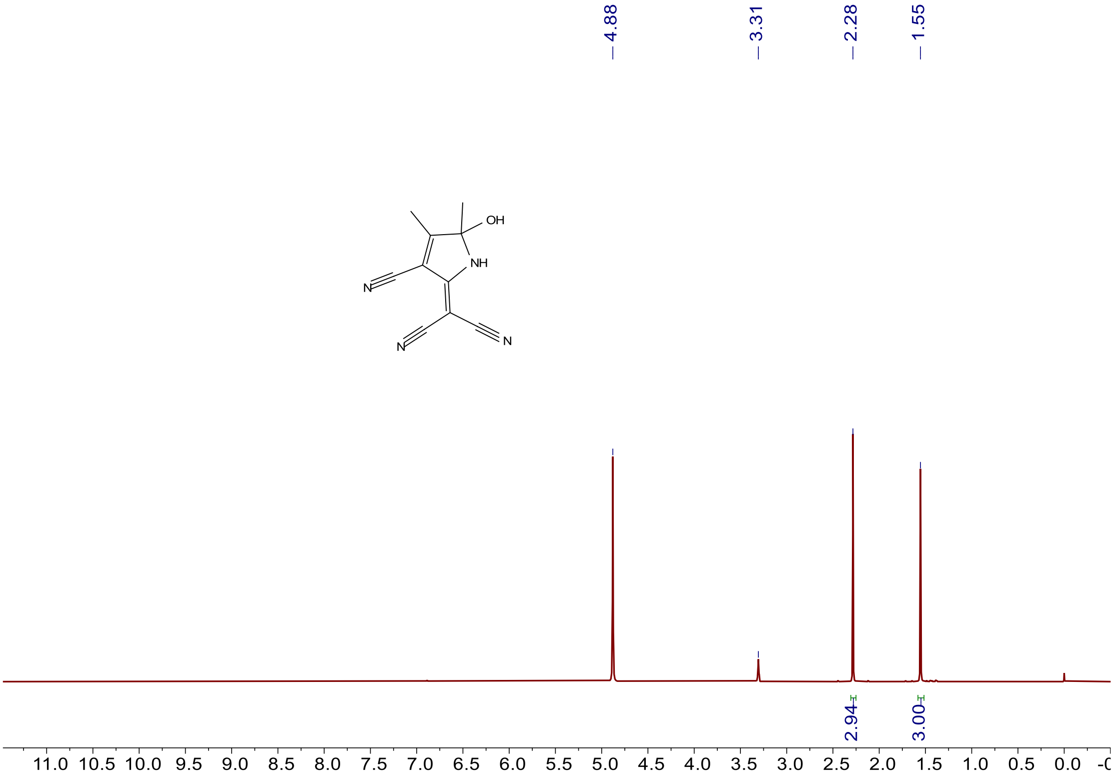

Figure S1 ^{1}H NMR spectrum of HTCP in CD_3OD

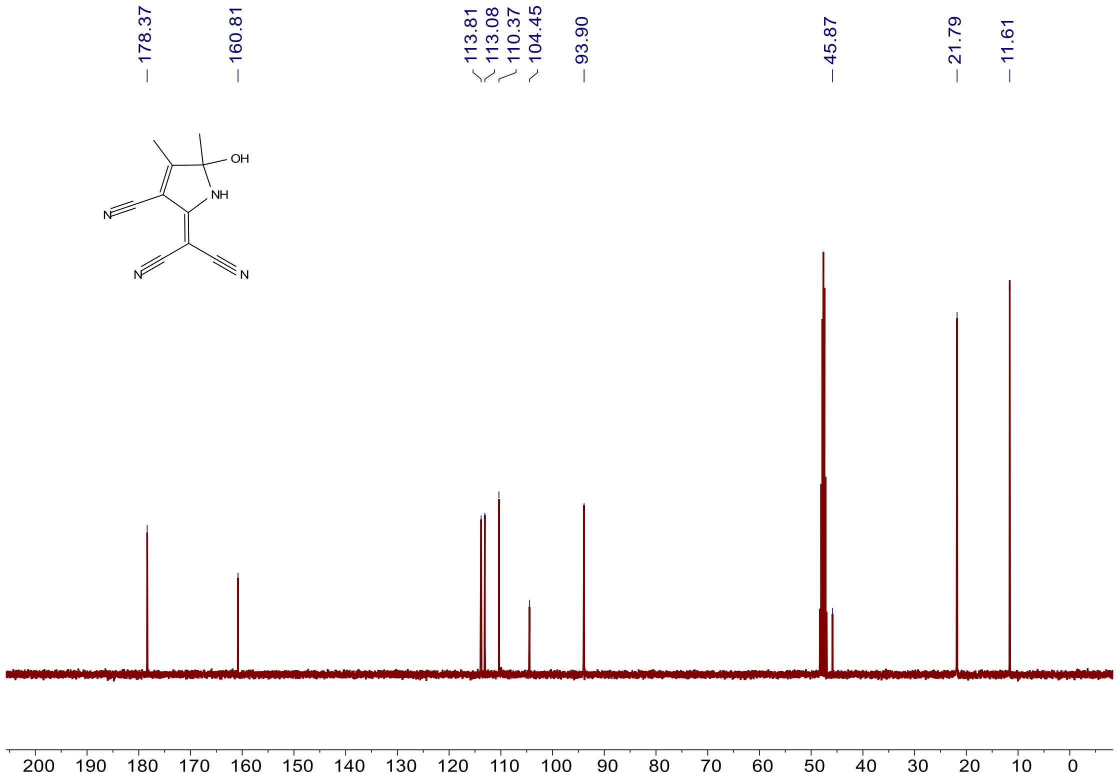

Figure S2 ^{13}C NMR spectrum of HTCP in CD_3OD

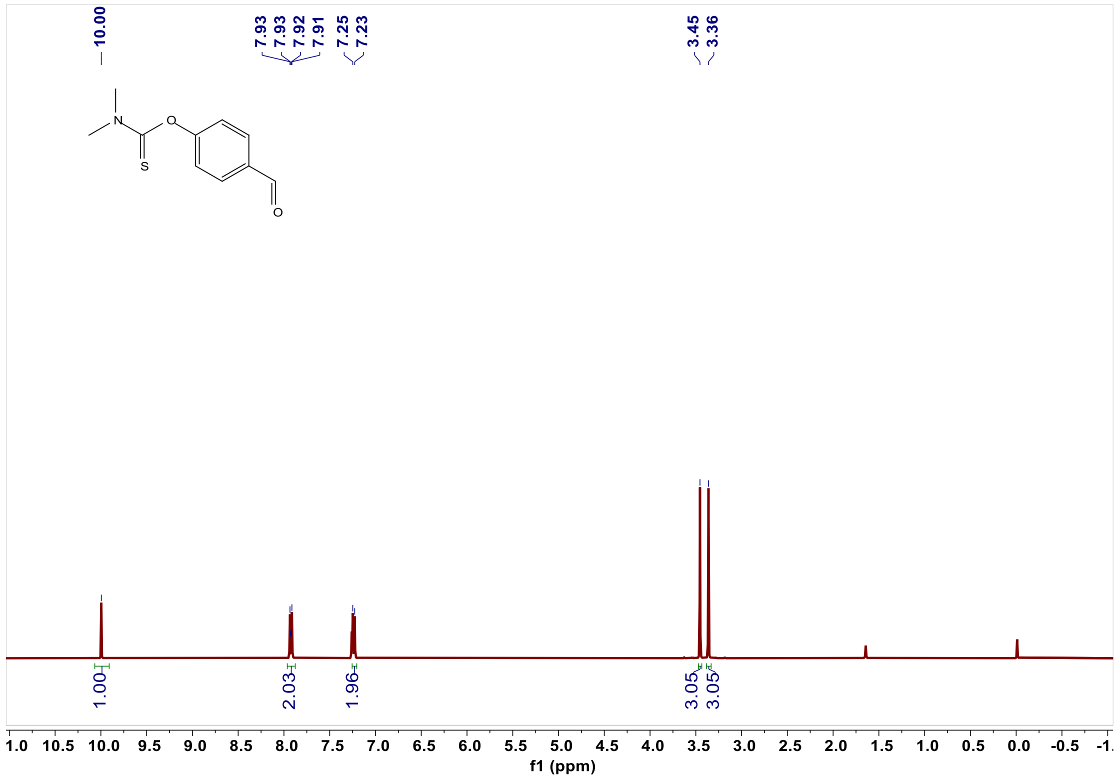

Figure S3 ^{1}H NMR spectrum of NTC in $CDCl_3$

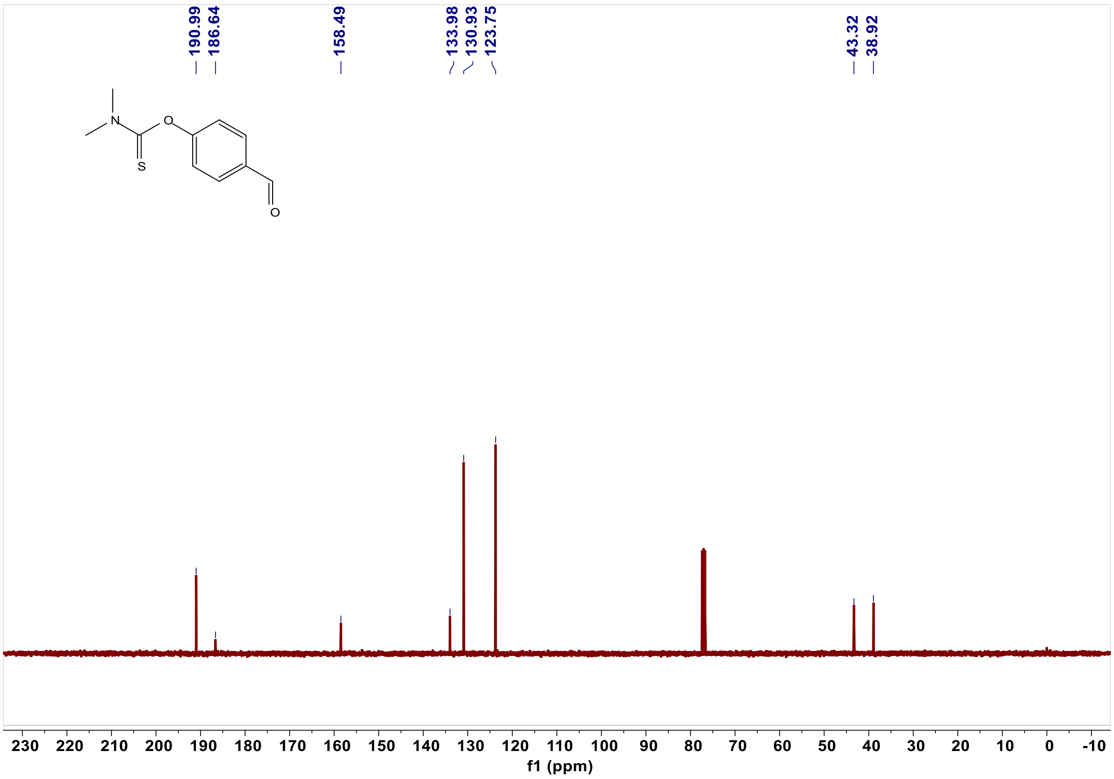

Figure S4 ^{13}C NMR spectrum of NTC in $CDCl_3$

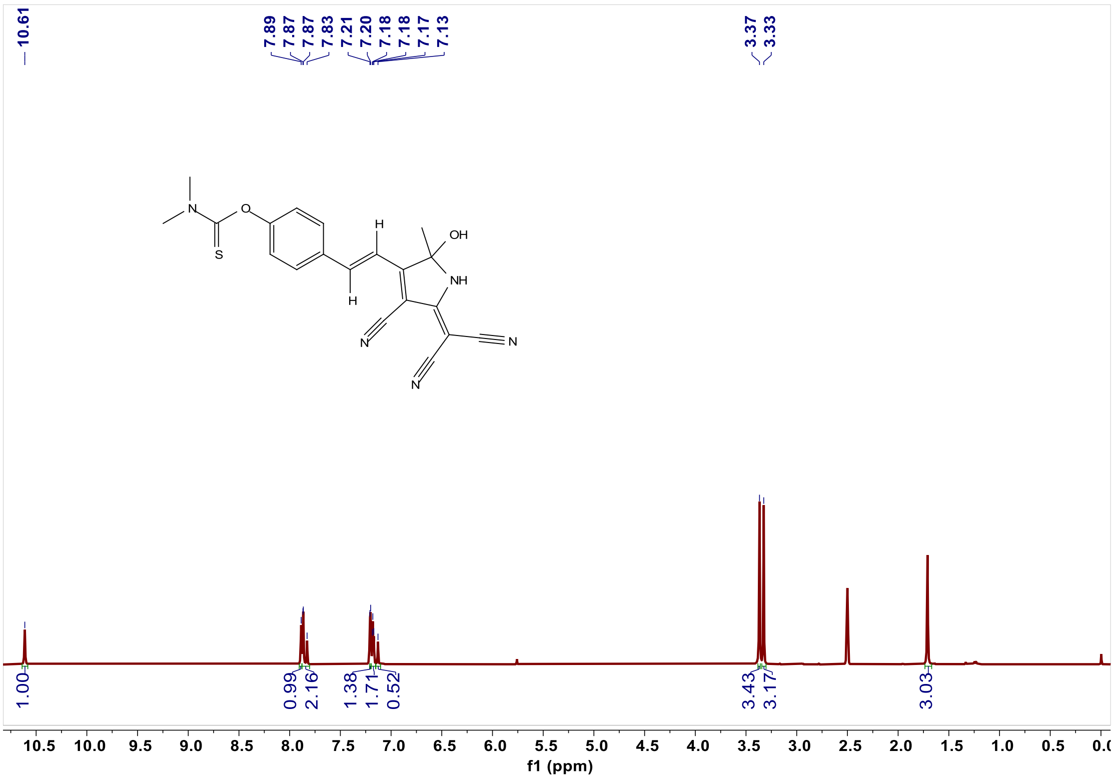

Figure S5 ^{1}H NMR spectrum of HTCP-NTC in DMSO-d_6

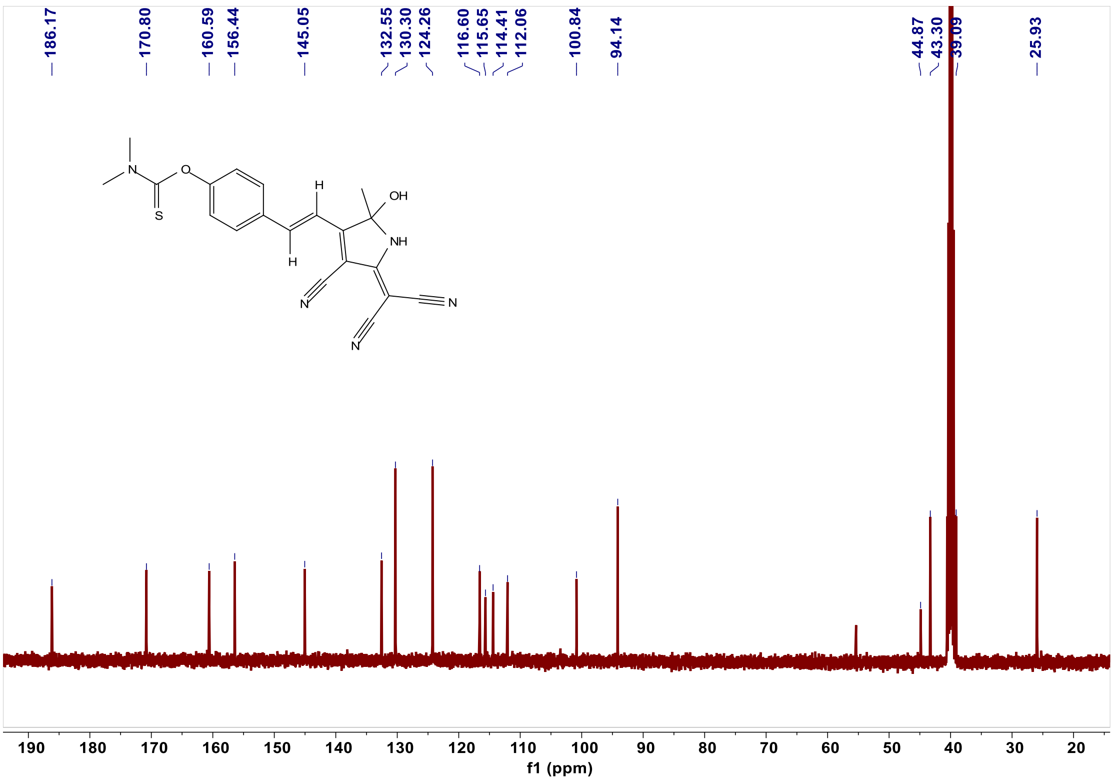

Figure S6 ^{13}C NMR spectrum of HTCP-NTC in DMSO-d_6

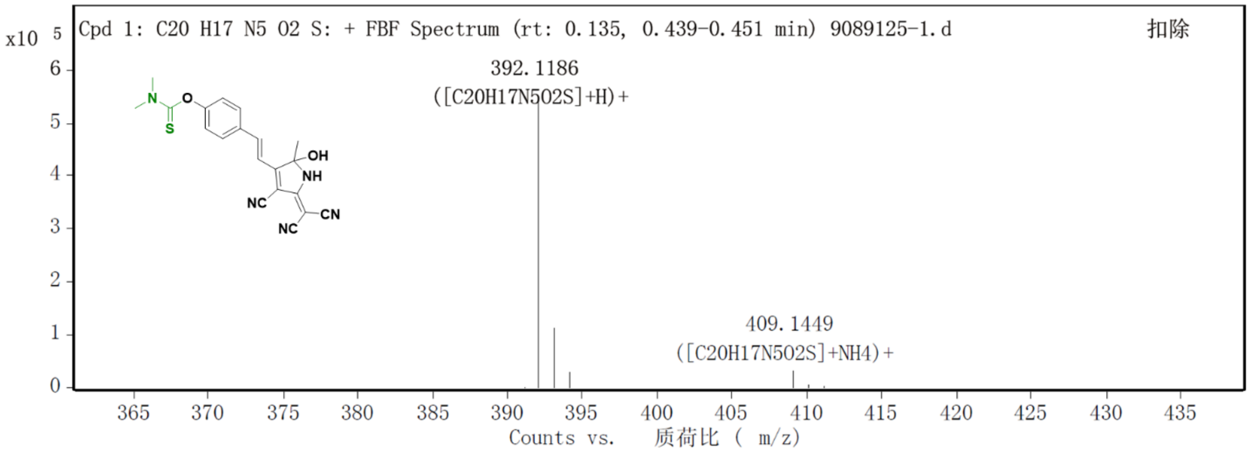

Figure S7 ESI-Mass spectrum of HTCP-NTC

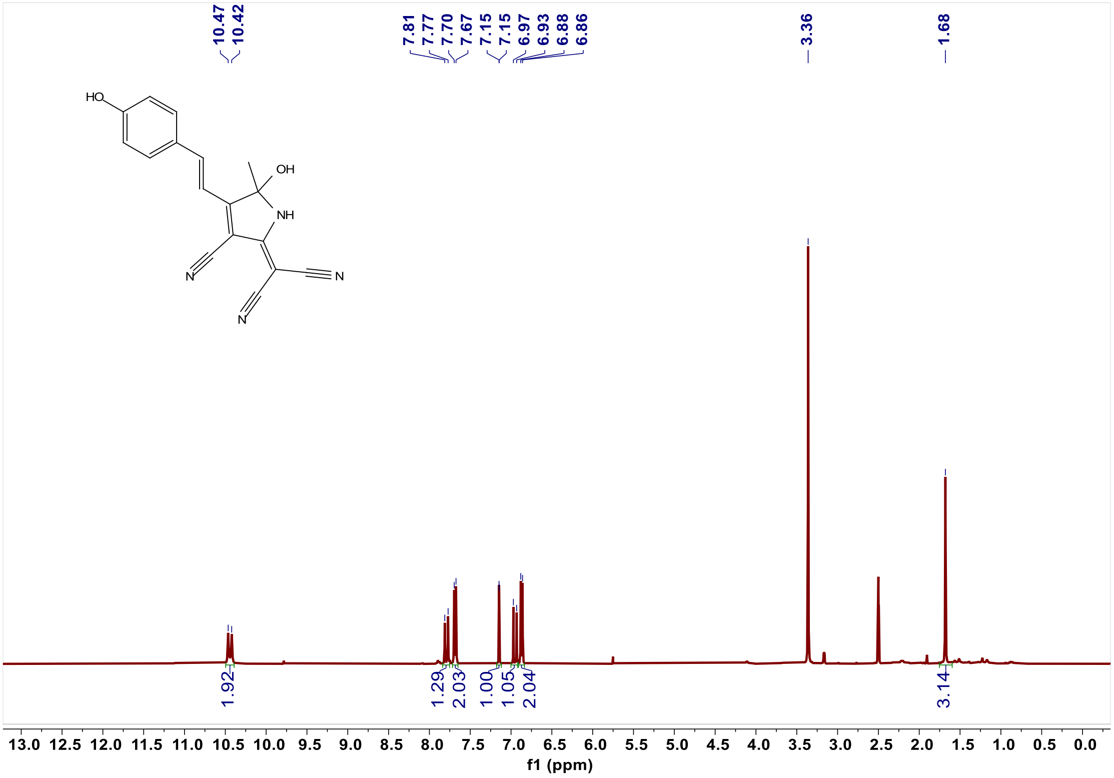

Figure S8 ^{1}H NMR spectrum of HTCP-OH in DMSO-d_6

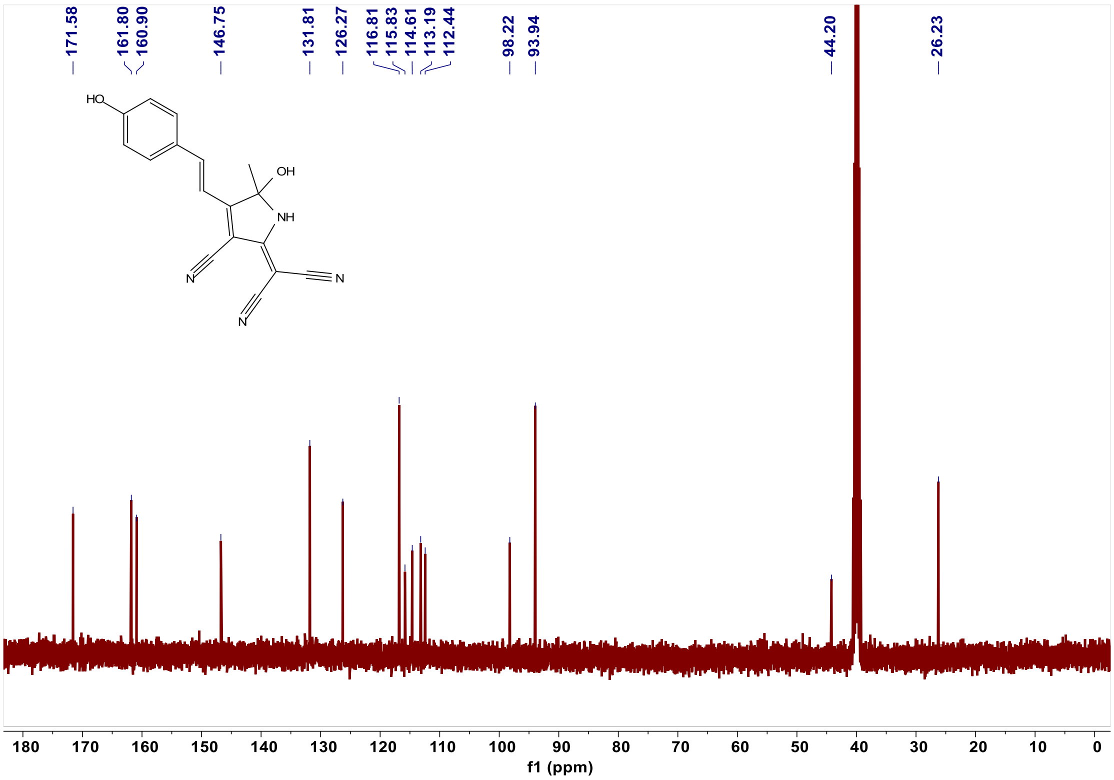

Figure S9 ^{13}C NMR spectrum of HTCP-OH in DMSO-d_6

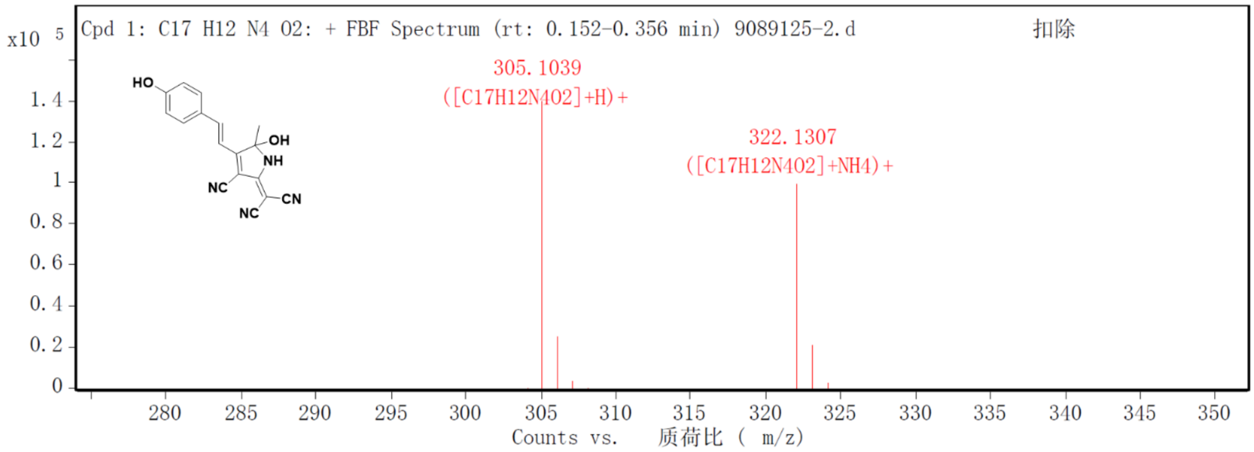

Figure S10 ESI-Mass spectrum of the compound HTCP-OH

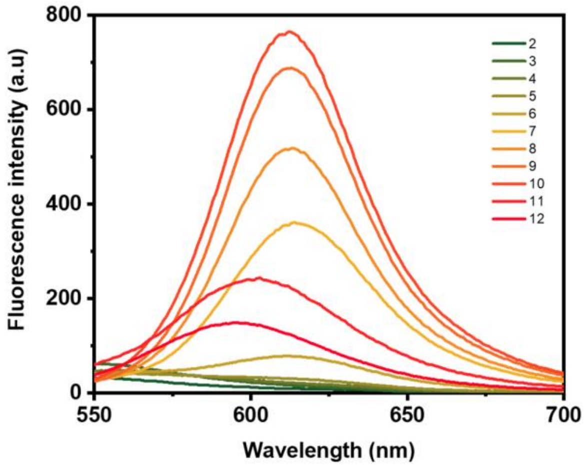

Figure S11 Fluorescence emission spectra of HTCP-NTC (10 μM) with HOCl (20 μM) in PBS buffer solution (10 mM, containing 1% EtOH) at different pH. (λ_{ex} = 520 nm)

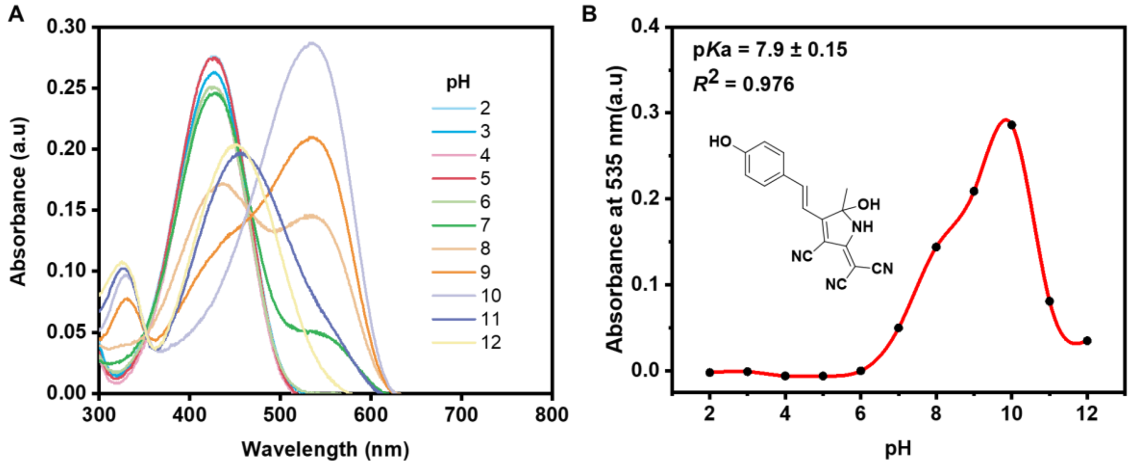

Figure S12 (A) The absorbance changes at different pH (2-12) for HTCP-OH. (B) The p*K*a fitting curve of HTCP-OH.

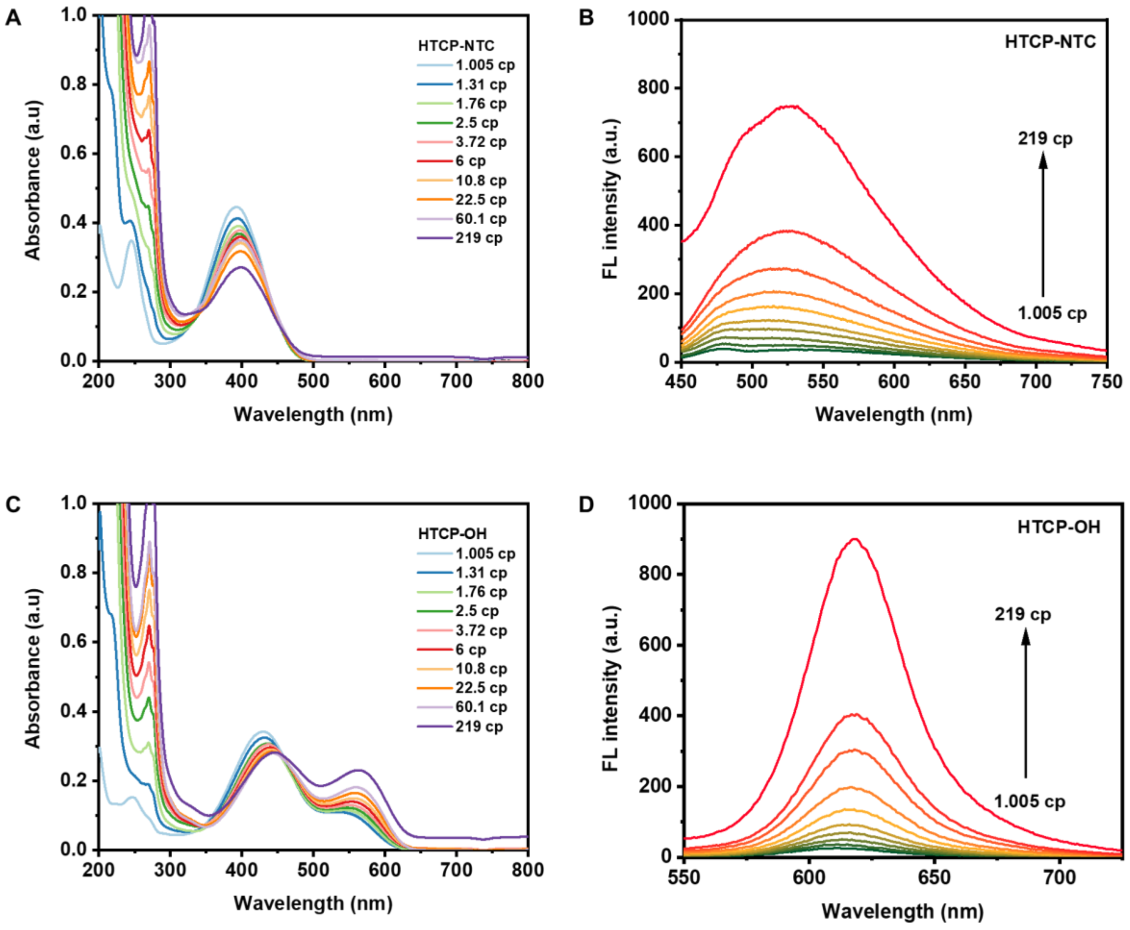

Figure S13 The absorption and fluorescence changes for HTCP-NTC (10 μM) (A, B) and HTCP-OH (10 μM) (C, D) in various viscosity (PBS 7.4/glycerol system).

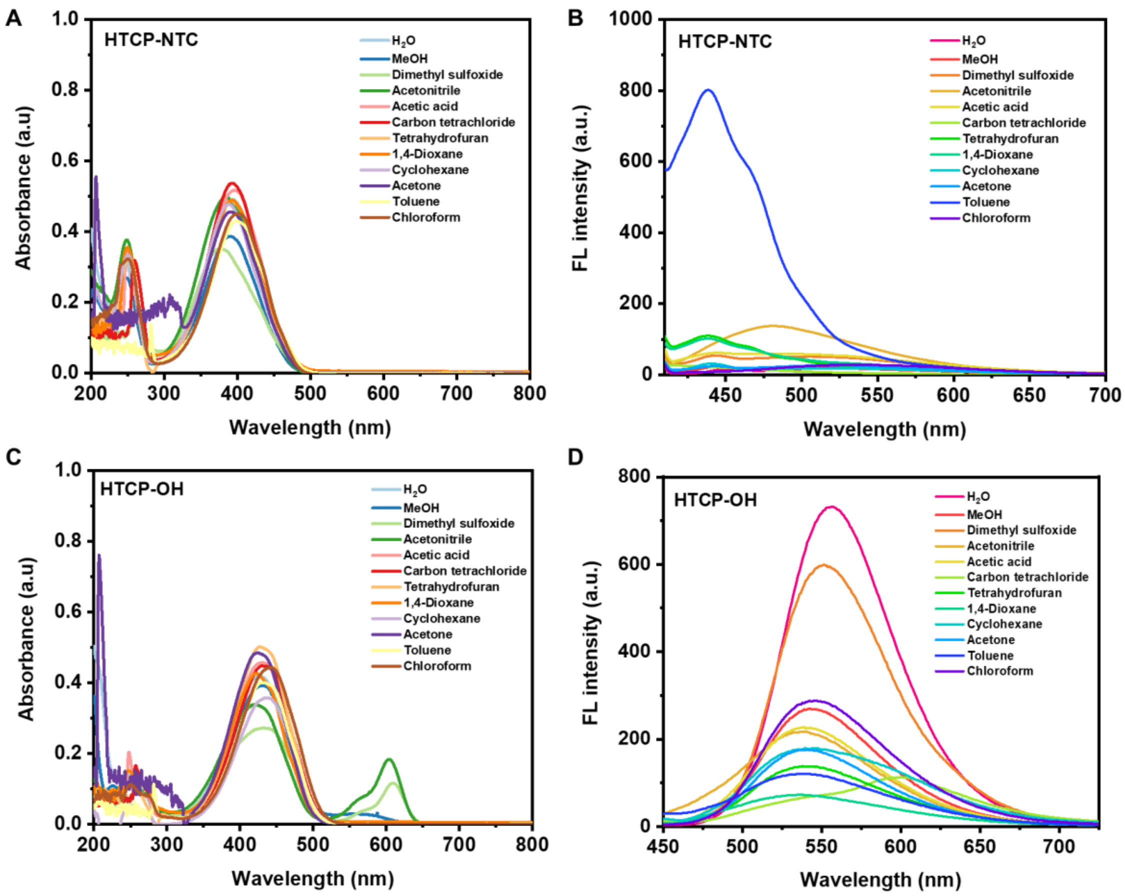

Figure S14 The absorption and fluorescence changes for HTCP-NTC (10 μM) (A, B) and HTCP-OH (10 μM) (C, D) in solvents of different polarity.

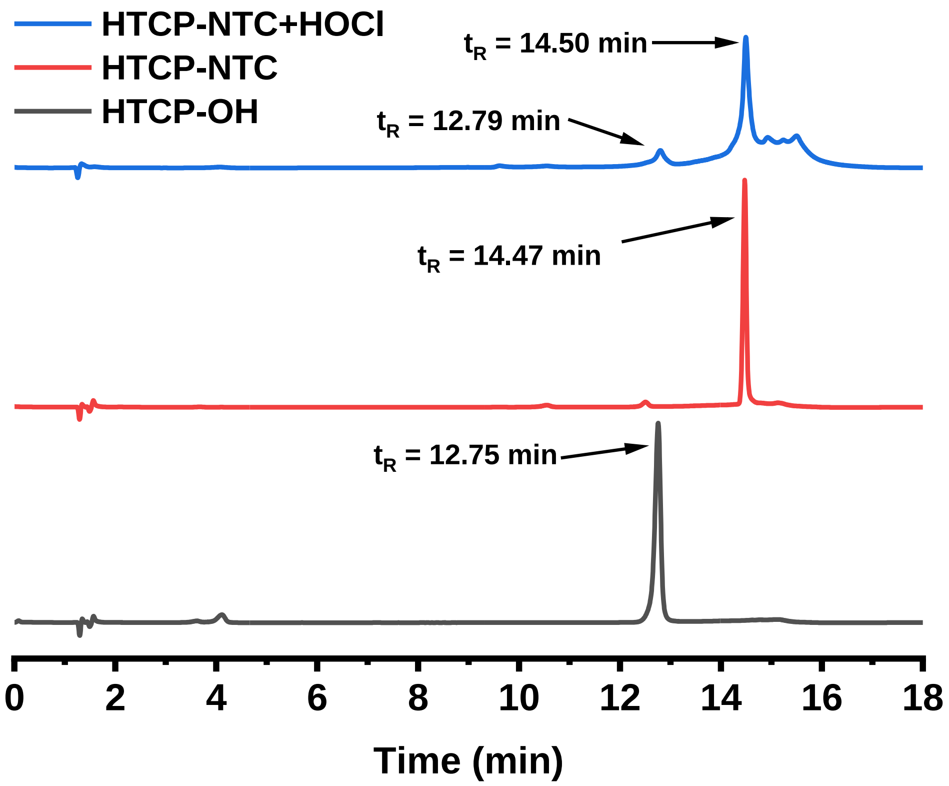

Figure S15 HPLC analysis of HTCP-NTC, HTCP-OH, and the reaction solution of HTCP-NTC incubated with HOCl for 15 min.

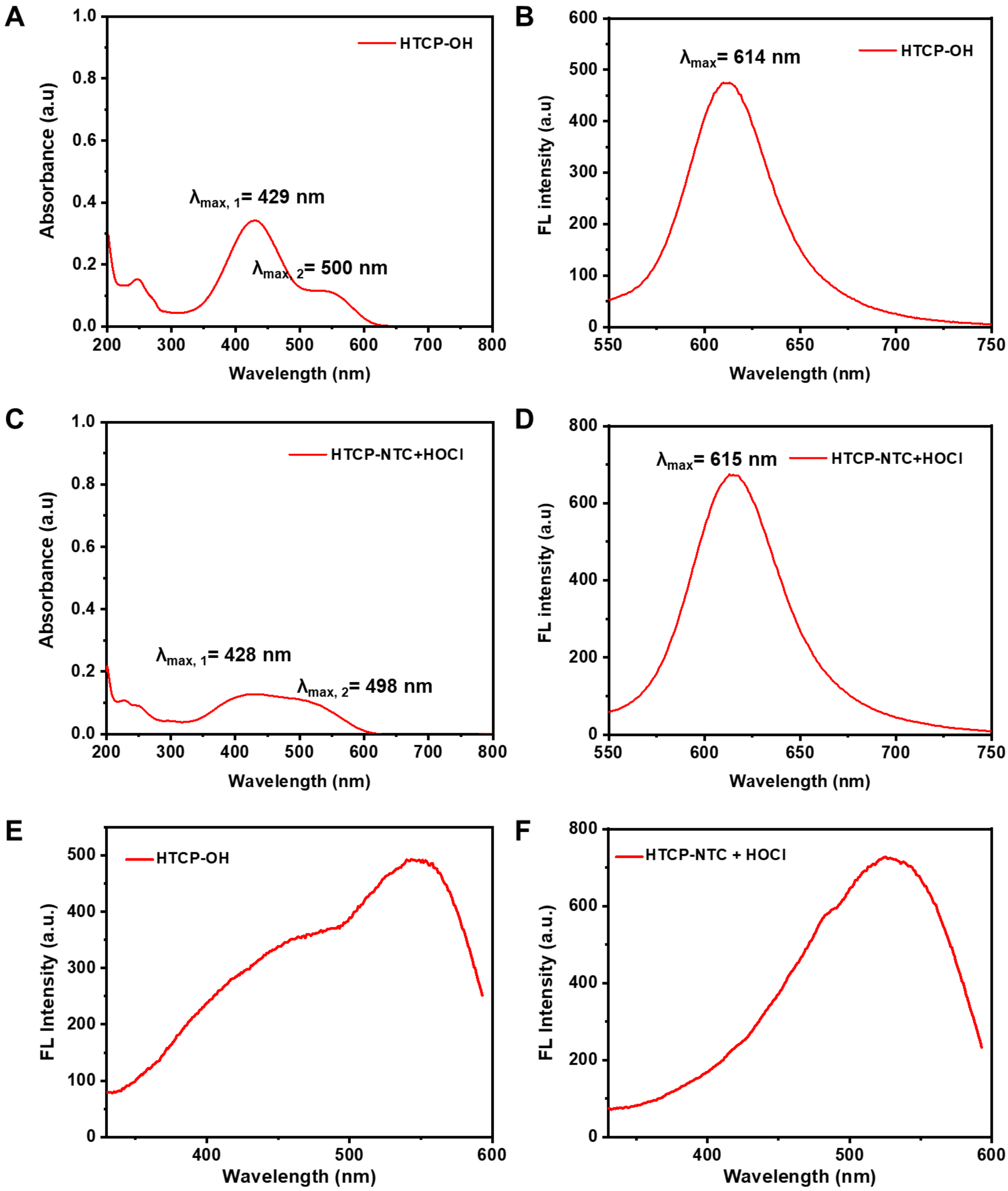

Figure S16 The absorption, fluorescence emission and excitation spectra of HTCP-OH (A, B, and E) and the reaction mixtures for HTCP-NTC with HOCl (C, D, and F).

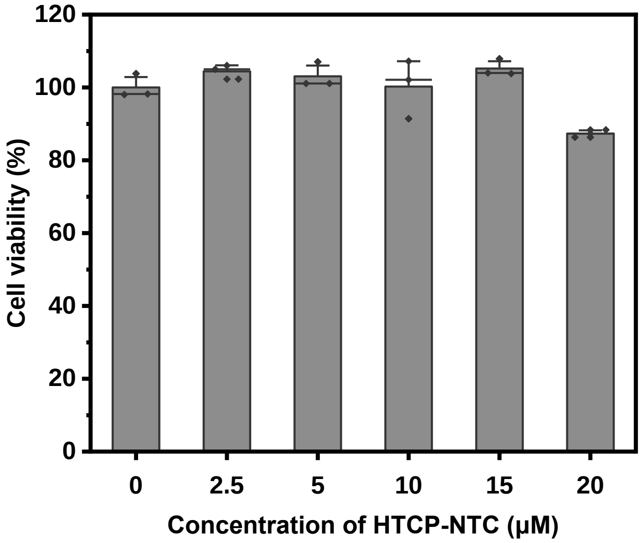

Figure S17 The cell viability of RAW264.7 after 24 h incubation with HTCP-NTC (Mean ± SD, n = 3)

Table S1 The summary of photophysical properties of reported similar probes

Probe	λ_{ex} (nm)	λ_{em} (nm)	Stokes shift (nm)	Detection time	Response type	LOD	Ref.
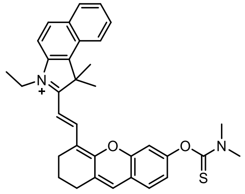	650 nm	732 nm	82	30 s	Off-On	35 nM	[1]
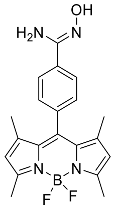	500	529	29	With 60 s	Off-On	6.81 μM	[2]
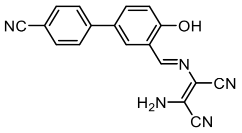	386/325	607/500	175	30 s	Ratiometric	3.34×10^{-7} M	[3]
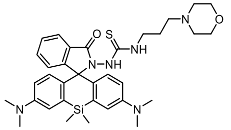	660	680	20	4 min	Off-On	20 nm	[4]
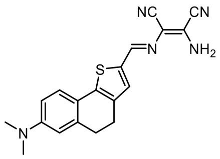	475	500	75	Within 60s	Off-On	0.15 μM	[5]

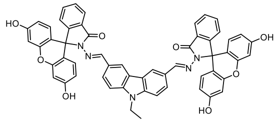	470	530	60	within 1.5 min	Turn-on	0.056μM	[6]
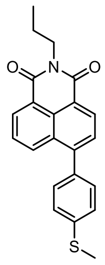	378/362	500/432	70	within 2 min	Ratiometric	0.02 μM	[7]
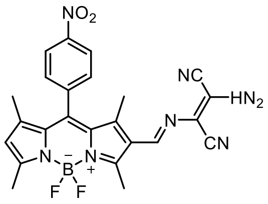	495	525	30	110 min	Turn-on	0.27 μM	[8]
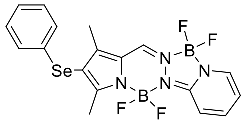	413	437	24	100 s	Turn-on	0.15μM	[9]

References

[1] Qian X, Yu H, Zhu W, et al. Near infrared fluorescent probe for in vivo bioimaging of endogenous hypochlorous acid[J]. Dyes and Pigments, 2021, 188:109218. DOI: 10.1016/j.dyepig.2021.109218.

[2] Wang L, Li B, Jiang C, et al. A BODIPY Based Fluorescent Probe for the Rapid Detection of Hypochlorite[J]. Journal of Fluorescence, 2018, 28(4):933–941. DOI: 10.1007/s10895-018-2255-y.

[3] Tang X, Zhu Z, Liu R, et al. A novel ratiometric and colorimetric fluorescent probe for hypochlorite based on cyanobiphenyl and its applications[J]. Spectrochimica acta. Part A, Molecular and Biomolecular Spectroscopy, 2019, 219:576–581. DOI: 10.1016/j.saa.2019.04.042.

[4] Mao G-J, Liang Z-Z, Bi J, et al. A near-infrared fluorescent probe based on photostable Si-rhodamine for imaging hypochlorous acid during lysosome-involved inflammatory response[J]. Analytica Chimica Acta, 2019, 1048:143–153. DOI: 10.1016/j.aca.2018.10.014.

[5] Ning Y, Cui J, Lu Y, et al. De novo design and synthesis of a novel colorimetric fluorescent probe based on naphthalenone scaffold for selective detection of hypochlorite and its application in living cells[J]. Sensors and Actuators B: Chemical, 2018, 269:322–330. DOI: 10.1016/j.snb.2018.04.156.

[6] Wang N, Xu W, Song D, et al. A fluorescein-carbazole-based fluorescent probe for imaging of endogenous hypochlorite in living cells and zebrafish[J]. Spectrochimica Acta. Part A, Molecular and Biomolecular Spectroscopy, 2020, 227:117692. DOI: 10.1016/j.saa.2019.117692.

[7] Xu C, Wu T, Duan L, et al. A naphthalimide-derived hypochlorite fluorescent probe from ACQ to AIE effect transformation[J]. Chemical Communications, 2021, 57(86):11366–11369. DOI: 10.1039/d1cc04157f.

[8] Cheng W, Ren C, Liu S, et al. A highly selective A–π–A “turn-on” fluorescent probe for hypochlorite in tap water[J]. New Journal of Chemistry, 2022. DOI: 10.1039/D2NJ01792J.

[9] Shelar D S, Malankar G S, M. M, et al. Selective detection of hypochlorous acid in living cervical cancer cells with an organoselenium-based BOPPY probe[J]. New Journal of Chemistry, 2022, 46(36):17610–17618. DOI: 10.1039/D2NJ02956A.
